# A retrospective analysis of substance use among female psychiatric patients in Saudi Arabia

**DOI:** 10.3389/fpsyg.2022.843785

**Published:** 2022-08-22

**Authors:** Abdulaziz A. Alodhayani, Khalid M. Almutairi, Jason M. Vinluan, Wadi B. Alonazi, Hatim Gormallah Alzahrani, Mohammed Ali Batais, Fatmah Mohammed Kaki, Turky H. Almigbal, Saad Alsaad

**Affiliations:** ^1^Department of Family Medicine and Community, College of Medicine, King Saud University, Riyadh, Saudi Arabia; ^2^Department of Family Medicine, Prince Sultan Military Medical City, Riyadh, Saudi Arabia; ^3^College of Business Administration, King Saud University, Riyadh, Saudi Arabia; ^4^Mental Health Hospital, Taif, Saudi Arabia; ^5^Mental Health Hospital, Ministry of Health, Jeddah, Saudi Arabia

**Keywords:** drug abuse, Saudi Arabia, retrospective study, female, psychiatric patients

## Abstract

**Objective:**

This study investigated the prevalence of substance use (SU), and its risk factors, among women attending psychiatric outpatients center in Saudi Arabia.

**Design:**

A retrospective cross-sectional design.

**Materials and methods:**

We reviewed outpatients’ records of 200 female patients with a history of SU from a psychiatric unit in Jeddah, Saudi Arabia from December 2018 to February 2019. The researchers developed the *pro forma*, and 2 psychiatrists and a family medicine physician validated the form.

**Results:**

The most common and widely used were psychoactive substances (58%), followed by central nervous system (CNS) depressants (22%), and finally cannabinols (9.5%). Overall, the highest substance use was the amphetamine-cannabis-nicotine (ACN) representing nearly half of the illicit items (46.6%), followed by heroine-alcohol-benzodiazepine (16.4%), and with the lowest being benzodiazepine-nicotine (1.7%). There was a significant difference between the single substance and multiple substance use in terms of age (*p* = 0.001), smoking behavior (*p* = 0.001), patients past history (*p* = 0.005), and age of the patient at the start of drug use (*p* = 0.005).

**Conclusion:**

Although the prevalence of substance use among women is low in Saudi Arabia, screening of substance use disorders risks and building a rehabilitation program to control drug dependence are needed.

## Introduction

Substance use (SU) and its consequences are major global concerns with long-term and immediate personal, social, and health impacts. Alcohol use and other SU (encompassing prescribed drugs) are costly, common worldwide, and have a global burden (GBD 2016 Alcohol and drug use collaborators, 2018). It is evident that SU is considered one of the public health concerns worldwide. In Arab countries, several studies show the prevalence of SU is rising, including Saudi Arabia (Alblooshi et al., 2016; Ibrahim et al., 2018; Al-Alawi and Shaikh, 2018). Apparently, recent evidence shows the association of SU among persons with mental illness (Nahas et al., 2017; Nielsen et al., 2017). Studies show that the prevalence of SU in persons with mental illness has higher rates compared with the general population and in particularly of amphetamines, alcohol and cannabis (Nielsen et al., 2017). Studies have also claimed that gender differs distinctively in SU etiology (Baggett et al., 2015; Verona et al., 2015). Various studies have found that men have higher rates of drug dependence and substance use than women who have rates that are three- and five-times lower, and women are less likely to concomitantly use cocaine, alcohol, and tobacco (Cowlishaw and Hakes, 2015; Zakiniaeiz and Potenza, 2018). Even though the rates of substance use are currently lower among women than in men, recent epidemiological research shows that SU among women is increasing (McHugh et al., 2014; Sweileh et al., 2014). The fact that SU is gendered and research on SU is more focused on men suggests that there are several biological, psychological, and social differences that may influence the development and treatment of SU between men and women (McHugh et al., 2018).

Multiple factors that may contribute to the development of a variety of short- and long-term SU and dependence include parental violence, a family member who used drugs, child sexual abuse, being male, younger or adolescent, and low educational attainment (Tahiraj et al., 2016; Narain et al., 2020). The comorbidity of depression and smoking history is revealed to be more prevalent in women than in men (Kim et al., 2016). Depression and stress are strongly associated with cigarette smoking in population studies (Flurhurty et al., 2017). Other factors, such as history of child abuse, should also be clearly differentiated between male and female treatment plans because they are significantly associated with substance use (Anderberg and Dahlberg, 2018). Furthermore, a cross-sectional study exploring factors associated with drug and alcohol dependence in people with severe mental illness in Italy, revealed that women were more likely to suffer from dependence in opioids and prescription drugs (Carrà et al., 2015). Identification of factors associated with substance use and dependence may help clinicians in targeting and managing patients who needs to receive appropriate treatment. In view of the recent literature about SU among patients with disorders and its gender-specific difference, it is important for the present study to recognize that sex is a significant factor in drug abuse. In addition, it is substantial to rely on the study outcomes implications and the increasing trend of SU among female psychiatric patients. Other psychological factors, such as depression, anxiety, self-destructiveness, suicidal behavior, low self-esteem, and difficulties with interpersonal communications might also contribute to the development of a variety of short- and long-term SU and dependence (Anderberg and Dahlberg, 2018; Narain et al., 2020).

Based on the World Drug Report 2014 conducted by the World Health Organization (WHO), approximately 5.25% of the world’s adult population, approximately 243 million, used illicit drugs, such as cannabis, opioids, and cocaine or amphetamine-type stimulants in 2012 (World Health Organization (WHO), 2014). Various studies show the prevalence of alcohol and SU rates in the Arab Gulf region among patients with psychiatric disorders (Jaalouk et al., 2012; Bajwa et al., 2013). A research study conducted in Kuwait revealed the lifetime prevalence of illicit drug use was 14.4% among male public and private students (Bajwa et al., 2013). In Saudi Arabia, a retrospective study of identified polysubstance abuse and amphetamine was the most predominant among 20–40 years old among patients admitted for substance abuse in a psychiatric hospital in Al-Qassim (Ibrahim et al., 2018). A retrospective study reported that a small sample of women attending a psychiatric setting in Saudi Arabia had polysubstance drug use (54%) including heroin (27.5%) (Algamdi and Ibrahim, 2013).

Over the years, understanding the nature of the most frequent SU in a specific gender is helpful for both healthcare policymakers and physicians (McHugh et al., 2018; Arnold et al., 2021). Identifying the patterns of SU among psychiatric female patients may help healthcare policymakers and healthcare providers to establish effective treatments and rehabilitation programs. In addition, there is a scarcity of studies addressing women’s SU disorders in Arab families and the Gulf countries in particular. This study was designed to determine the prevalence of substance abuse among psychiatric female patients and classify the most commonly used ones according to their socio-demographic features.

## Materials and methods

### Design and setting

We conducted a retrospective analysis among patients with a history of SU in a major psychiatric hospital in Jeddah, Saudi Arabia, from December 2018 to February 2019. The selected hospital was chosen because it provides wide range of mental health services, such as drug use treatment and rehabilitation as long-term care for patients seeking help. This hospital is also specialized in addiction management on a national level.

### Study sample

The inclusion criteria included all Saudi women aged 18 years old and above, involved with SU and currently being treated for only SU with regular follow-up at outpatient clinics. Patients under the age of 18 years old and those with missing data and/or insufficient data were excluded from the study. During the study period, a total of 274 records were listed with a history of substance use. Upon reviewing the medical records, 74 records were excluded due to missing and/or insufficient data.

### Measures and data collection

Initially, the researchers developed a *pro forma* and a panel of two psychiatrists and a family medicine physician validated it. It included the socio-demographic characteristics of the participants (age, marital status, educational level, occupation, income, and living status) as well as the pattern of SU (name of substances used, number and duration of SU, age at start of SU, and the last use of substances). In addition, the checklist contained additional information regarding smoking history, previous hospital admissions, source of SU, history of abuse (e.g., physical and emotional), and history of suicidal attempts. We piloted the *pro forma* with 15 medical records to ensure the appropriateness of the method and the availability of data.

All concerned patients officially consented to review their medical records for the study purpose. The Institutional Review Board Committee in the Jeddah psychiatry hospital granted a waiver of informed consent approval (IRB no: H-02-J-002). A comprehensive orientation about the objectives of the study was given by two investigators to the head of nursing and 2 specialist nurses. Liaisons from the nursing department were trained and they completed the checklist. To ensure the confidentiality of the obtained information, a survey package was distributed to the nurses; each package included the checklist in English and an A4 self-sealing envelope. Nurses were instructed to read and fill out the checklist from the medical records and to seal it inside the envelope once completed. To ensure inter-rater reliability, the head nurse re-checked the data. At the end of each week, one of the investigators collected all of the sealed envelopes. All of the records were coded anonymously and the data were kept confidential.

### Statistical analysis

To achieve the study objectives, we used an online sample size calculator with a minimum recommended sample size of 199. The Raosoft Sample Size Calculator calculated the sample using the following equation: *n* = z^2^P (1-P)/E^2^. The standard normal deviation is 99% (Z) and the confidential level is 2.04. The sampling error is at ± 1% = (0.01) (E), and *P* is the percentage picking a response at 50%, which is 0.5. Data were entered using SPSS version 21 for Windows (SPSS, Armonk, NY, United States) and analyzed using descriptive statistics, such as means, standard deviations (SDs), frequencies, and percentages. After reviewing the medical records, we compared the medical conditions, socio-demographic data, and the pattern of SU. Investigators created charts based on the study’s objectives with the help of a biostatistician. First, we categorized the substance used based on the types of drug: central nervous system (CNS) stimulants and depressants, Cannabinols, Opiates, Inhalants, and multiple psychoactive substances. Then, we dichotomize the substance into single substance use and multiple substance use. Pearson’s chi-square test was used to analyze the associations between 2 categorical variables. Logistic regression was done to assess the predictors associated with substance use of the participants. A *p*-value < 0.05 was considered statistically significant.

## Results

Table 1 presents the social demographic characteristics of the participants. Half of the participants were 31–40 years old. Together, slightly over half of the respondents were married or divorced. More than one-quarter was employed in the public and private sectors. Most of the respondents had no income (64.5%) and 89% lived with their families. The results of the chi-square analysis for each of the independent categorical variables are presented under *p*-values within 95% confidence intervals (*CI*s) and we classified them into three types of substances.

**TABLE 1:** Socio-demographic characteristics of female psychiatric patients with substance use (SU) (*n* = 200).

Characteristic	Variable	*N*	%
Age	18–30 years	72	36.0
	31–40 years	100	50.0
	41 and above	28	14
Marital status	Single	64	32.0
	Married	57	28.5
	Divorced	50	25.0
	Widowed	29	14.5
Educational level	Primary school	33	16.5
	Secondary school	91	45.5
	University degree	76	38.0
Employment status	Unemployed	128	64.0
	Employed	72	36
Income	No income	129	64.5
	Less than $1333*	21	10.5
	$1333–2666	31	15.5
	More than $2666	19	9.5
Living in	Urban area	104	52.0
	Rural area	96	48.0
Living with	Family	178	89.0
	Not with family	22	11.0
Smoking behavior	Yes	183	91
	No	17	9
Suicide attempt	Yes	114	57
	No	86	43
Patient’s past history	Medical illness	39	19
	Physical abuse	8	4
	Child abuse	28	14
	Psychological disorders	126	63
Age of the patient at start drug use	30 and below	171	85.5
	31–45	24	12
	46 and above	5	2.5

*3.75 Saudi Riyal = US $1.

As shown in Table 2, the most common and widely used were multiple psychoactive substances (58%), followed by CNS depressants (22%), and finally Cannabinols (9.5%). Overall, the highest SU was with amphetamine-cannabis-nicotine (ACN) representing nearly half of the illicit drugs (46.6%), followed by heroine-alcohol-benzodiazepines (16.4%), and with the lowest being benzodiazepine-nicotine (1.7%) (Table 3).

**TABLE 2:** Distribution of types of substance use among female psychiatric outpatients.

Psychoactive substance	Frequency	%
CNS stimulants	16	8
CNS depressants (Downers)	44	22
Cannabinols	19	9.5
Opiates	4	2
Inhalants	1	0.5
Multiple psychoactive substance use	116	58
Total	200	

**TABLE 3:** Multiple psychoactive substance use among female psychiatric outpatients.

Psychoactive substance	Frequency	%
Heroine-alcohol-benzodiazepine	19	16.4
Amphetamine-cannabis-benzodiazepine	17	14.7
Alcohol-benzodiazepine-nicotine	3	2.5
Cannabis-benzodiazepine-nicotine	18	15.5
Cannabis-nicotine	3	2.5
Benzodiazepine-nicotine	2	1.7
Amphetamine-cannabis-nicotine	54	46.6
Total	116	

As shown in Table 4, there was a significant difference between single substance use and multiple substance use in terms of age, smoking behavior, patients past history, and age of the patient at start of drug use. The intake of multiple substance was higher in patients aged 18–30 and 31–40, while single substance use was more common in patients aged 51–60 (*p* = 0.001). The analysis shows that there was a significant difference between smoking status and patients’ substance use. The majority of the smokers (63%) intake multiple SU while non-smokers were on single substance use only. With regards to the past history of patients, the majority of patients who had a medical illness (60%), physical abuse (87%), and child abuse (82%) were multiple substance users and half of the patients with psychological disorders take single substance use only (50%) (*p* = 0.005). There was a significant difference among the age of the patient at the start of drug use (*p* = 0.005).

**TABLE 4:** Difference between single and multiple substance use of the participants in relation to their sociodemographic characteristics.

Variable	SSC* *N* = 84 (%)	MSC^†^ *N* = 116 (%)	Value	d*f*	*P*-value
Age			17.253	2	**0.001**
18–30	18 (25)	54 (75)			
31–40	47 (47)	53 (53)			
51–60	19 (67.9)	9 (32.1)			
Marital status			0.113	1	0.428
Single	59 (41.3)	84 (58.7)			
Married	25 (43.9)	32 (56.1)			
Educational level			4.172	1	0.029
Primary - Secondary	59 (47.6)	65 (52.4)			
University degree	25 (32.9)	51 (67.7)			
Employment status			0.051	1	0.468
Unemployed	53 (41.4)	75 (58.6)			
Employed	31 (43.1)	41 (56.9)			
Income			0.060	1	0.463
No income	55 (42.6)	74 (57.4)			
With income	29 (40.8)	42 (59.2)			
Living in			0.232	1	0.367
Urban area	42 (40.4)	62 (59.6)			
Rural area	42 (43.8)	54 (56.3)			
Living with			1.597	1	0.150
Family	72 (40.4)	106 (59.6)			
Not with family	12 (54.5)	10 (45.5)			
Smoking behavior			20.717	1	**0.001**
Yes	68 (37.2)	115 (62.8)			
No	16 (94.1)	1 (5.9)			
Suicide attempt			0.695	1	0.245
Yes	45 (39.5)	69 (60.5)			
No	39 (45.3)	47 (54.7)			
Patients past history			12.968	3	**0.005**
Medical illness	15 (39.5)	23 (60.5)			
Physical abuse	1 (12.5)	7 (87.5)			
Child abuse	5 (17.9)	23 (82.1)			
Psychological disorders	63 (50)	63 (50)			
Age of the patient at start drug use			10.650	2	**0.005**
30 and below	65 (38)	106 (62)			
31–45	14 (58.3)	10 (41.7)			
46 and above	5 (100)	0			

Pearson’s chi-square test was used in this table, *SSC = patients who used a single substance; ^†^MSC = patients who used multiple substances. ^‡^p < 0.05. Bold values indicate statistical significance (p-value < 0.05).

Logistic regression was performed to assess the association between demographic factors and substance use of the participants. As shown in Table 5, only two independent variables made a unique contribution to the model (smoking behavior and past medical history). The strongest predictor of substance use was past medical history, particularly participants who had a history of physical abuse, recording an odds ratio (*OR*) of 12.91. This indicated that participants with a history of physical abuse were over twelve times more likely to use substances than those who did not have a medical history.

**TABLE 5:** Association between demographic factors and substance use of the participants using the logistic regression analysis.

Variable	OR	95% CI	*P*-value
Age			0.266
18–30	1		
31–40	0.70	0.29–1.69	
51–60	0.35	0.10–1.23	
Marital status			0.113
Single	1		
Married	0.92	0.39–2.16	0.849
Educational level			0.271
Primary - Secondary	1		
University degree	1.54	0.71–3.37	
Employment status			0.297
Unemployed	1		
Employed	0.37	0.06–2.34	
Income			0.172
No income	1		
With income	3.47	0.58–20.76	
Living in			0.753
Urban area	1		
Rural area	0.89	0.45–1.76	
Living with			0.808
Family	1		
Not with family	1.15	0.35–3.81	
Smoking behavior	0.25	0.01–0.24	**0.002**
Suicide attempt	0.78	0.39–1.55	0.492
Patients past history			**0.037**
Medical illness	1		
Physical abuse	12.91	0.38–43.55	
Child abuse	3.17	0.81–12.33	
Psychological disorders	0.96	0.30–1.57	
Age of the patient at start drug use			0.554
30 and below	1		
31–45	0.72	0.24–2.11	
46 and above	0.95	0.34–2.75	

CI, confidence interval; statistically associated at 0.05 level of significant. Bold values indicate statistical significance (p-value < 0.05).

## Discussion

Identifying the most common SU is a significant factor in preventing drug abuse and addiction as well as in formulating treatment strategies (Siddiqui and Salim, 2016). Evidence and scientific research are considered vital aspects in formulating treatment plans (Badr et al., 2014). Unfortunately, there is a scarcity of research about the prevalence of SU in Saudi Arabia, particularly among female patients. This study investigated the prevalence of the most common SU among female patients attending a psychiatric hospital in Saudi Arabia. The results of the study indicated that personal behaviors, such as tobacco smoking and presence of psychological disorders were the factors most associated with the type of SU among women (Berg et al., 2013; DeRuiter et al., 2013; Kelly et al., 2013). Unlike other countries where cocaine, heroin, and methadone are common, the results revealed that the most dominant SU among female patients was multiple psychoactive substance use (58%) followed by CNS depressants (22%), and the prevalence of opiates was only 2%.

The findings of the study indicate that 58% of patients use several substances (polysubstance use). Amphetamine-cannabis-nicotine (46.6%) is the major illicit substance used by the women in this study. The combined use of three or more substances or multiple substances use showed augmented effects or adverse effects on the brain structure as well as the functions of substance users (Licata and Renshaw, 2010; Noyan et al., 2016). A recent study shows abnormalities in the gray matter of the brain among polysubstance users (Noyan et al., 2016). Only a few studies have found the effects of polysubstance use or combination caused by amphetamines and other substances. However, previous reports accentuate polysubstance users’ rehabilitation (Zambon et al., 2017). A treatment strategy that is safe and effective for polysubstance users, such as combined multimodal treatment (family therapy, social rehabilitation, medications, and counseling) may paid off considering these circumstances.

Unlike many other healthcare systems, that of Saudi Arabia hospital culture, which comes from the general community culture, seems to encourage SU patients to be confidentially treated only in specialized public psychiatric centers. Therefore, this low rate when compared with other convicted SU women might be attributed to the need for both effective health promotion in Saudi society as well as exploring the role of religion and person-specific culture in people’s lives (Chen and Gueta, 2015). The study findings indicate more amphetamine use and probably more available for hands-on the street. Official reports have revealed that amphetamine seizures in Saudi Arabia decreased significantly from approximately 17,000 kg in 2009 to 11,000 kg in 2012 (World Drug Report 2014). Although there is a tendency toward a low level of SU among women worldwide, patient characteristics are good indicators for addiction. While the outcomes of the current study are similar to the research conducted by Sawwaf and Chaleby in terms of demographic characteristics and the psychiatric setting, the patients’ patterns varied (Sawwaf and Chaleby, 2018). As the results of this study suggest that past history and smoking behaviors were the patterns most associated with SU among the women attending the psychiatric center. Additionally, the results indicated that there were significant relations between SU and smoking habits, which is consistent with major research studies (Kelly et al., 2013; Weinberger et al., 2017). The high percentage of SU patients who are smokers supports the notion that there is still a relationship between smoking and SU, suggesting the need for cigarette-smoking cessation campaigns (Luger et al., 2014).

Comorbidity with psychological disorders put women to be more vulnerable to SU than patients with a history of medical illness, physical abuse, or child abuse, and they represented a high proportion in this study (Abdar Esfahani and Sayehmiri, 2014). In this study, more than half of the participants attempted suicide, which may indicate a negative effect of the substances and may increase the chance of developing psychiatric disorders, such as depression, loneliness, hopelessness, and anxiety. The person relationship problems, the presence of physical and emotional abuse, and psychological problems related to childhood can play a major role in explaining such a high percentage of SU behavior.

This study acknowledges some limitations, such as further studies are needed that include larger samples. The missing data and inappropriate interpretation of the medical documentation were concerns during the study. While we collected the data from only one psychiatric setting, the data cannot be generalized. In addition, there is a need for investigating the impact of both prescribed and illicitly obtained drugs to understand the holistic impact of SU, especially in clinical practice. However, this study offers vital information that can be used as a baseline finding since there is a scarcity of studies addressing women’s SU disorders in Gulf countries. Furthermore, this study can be used by policymakers in creating health education and health interventions addressing SU in Saudi Arabia as well as other countries.

According to the results, amphetamine-cannabis-benzodiazepine was the most frequently used and abused substance by women in Saudi Arabia. The age of patients, smoking behavior, past history, and age of the patient at starting drug use were found to be significant factors in single and multiple substance use of patients. Although the prevalence of female substance use is low in Saudi Arabia, further integrative programs are needed to control drug dependence. Promoting a rehabilitation program may control drug dependence as well as to increase awareness about the negative social and health impact of SU. Identifying the prevalence and risk factors associated with SU with women attending psychiatric outpatients center is important to better understand their needs and give interest to policymakers and future researchers in which area to be focus greater attention.

## Data availability statement

The data set used is locked and stored in the College of Applied Medical Science at King Saud University and can be obtained from the principal investigator on reasonable request.

## Ethics statement

The studies involving human participants were reviewed and approved by the Institutional Review Board Committee in the Jeddah Psychiatric Unit. Written informed consent for participation was not required for this study in accordance with the national legislation and the institutional requirements.

## Author contributions

All authors contributed to data analysis, interpretation of results, drafting or revising the manuscript, and agree to be accountable for all aspects of the work. All authors contributed to the article and approved the submitted version.

## Abbreviations

SU: substance use
WHO: World Health Organization
SSC: single substance content
ACN: amphetamine-cannabis-nicotine.
